# The Glycine-Rich RNA-Binding Protein Is a Vital Post-Transcriptional Regulator in Crops

**DOI:** 10.3390/plants12193504

**Published:** 2023-10-09

**Authors:** Ke Cheng, Chunjiao Zhang, Yao Lu, Jinyan Li, Hui Tang, Liqun Ma, Hongliang Zhu

**Affiliations:** 1 The College of Food Science and Nutritional Engineering, China Agricultural University, Beijing 100083, China; bs20223060553@cau.edu.cn (K.C.); b20213060520@cau.edu.cn (Y.L.); sy20193061114@cau.edu.cn (J.L.); s20213060979@cau.edu.cn (H.T.); lqma@cau.edu.cn (L.M.); 2 Supervision, Inspection & Testing Center of Agricultural Products Quality, Ministry of Agriculture and Rural Affairs, Beijing 100083, China; cc15809181@163.com

**Keywords:** glycine-rich RNA-binding protein, post-transcriptional regulation, plant development, stress response, liquid–liquid phase separation, crops

## Abstract

Glycine-rich RNA binding proteins (GR-RBPs), a branch of RNA binding proteins (RBPs), play integral roles in regulating various aspects of RNA metabolism regulation, such as RNA processing, transport, localization, translation, and stability, and ultimately regulate gene expression and cell fate. However, our current understanding of GR-RBPs has predominantly been centered on Arabidopsis thaliana, a model plant for investigating plant growth and development. Nonetheless, an increasing body of literature has emerged in recent years, shedding light on the presence and functions of GRPs in diverse crop species. In this review, we not only delineate the distinctive structural domains of plant GR-RBPs but also elucidate several contemporary mechanisms of GR-RBPs in the post-transcriptional regulation of RNA. These mechanisms encompass intricate processes, including RNA alternative splicing, polyadenylation, miRNA biogenesis, phase separation, and RNA translation. Furthermore, we offer an exhaustive synthesis of the diverse roles that GR-RBPs fulfill within crop plants. Our overarching objective is to provide researchers and practitioners in the field of agricultural genetics with valuable insights that may inform and guide the application of plant genetic engineering for enhanced crop development and sustainable agriculture.

## 1. Introduction

Gene expression in plants is typically subject to meticulous regulation at both the transcriptional and post-transcriptional strata. This intricate regulation assumes paramount importance, as it governs the growth and developmental processes of these organisms, while concurrently facilitating their ability to respond to and adapt to a myriad of environmental stimuli. RNA-binding proteins (RBPs) are central regulatory factors controlling post-transcriptional RNA metabolism during plant growth, development, and stress responses [1,2].

RBPs, a general term denoting ubiquitous proteins with the capacity to bind to RNA, interact with RNAs to form dynamic ribonucleoprotein (RNP), thereby assuming a central role in overseeing the destiny and functionality of RNA throughout its intricate life cycle [3]. As such, RBPs serve as pivotal orchestrators in the realm of gene expression by governing various facets, including RNA synthesis, processing (involving capping, splicing, and polyadenylation), editing, transportation, storage, surveillance, quality control, functionality, translation, and eventual RNA turnover. It is pertinent to note that modifications in RNA structure and spatial conformation may induce alterations in the specific RBPs bound to it, consequently giving rise to divergent biological functions [4]. Broadly, RBPs are characterized by the presence of putative RNA-binding domains, including, but not limited to, RNA recognition motifs (RRMs), K homology domains, zinc fingers, DEAD/DEAH boxes, Pumilio/FBF domains, and pentatricopeptide repeat (PPR) domains. Moreover, plant RBPs feature modular auxiliary domains rich in glycine, arginine, and serine residues, presenting diverse configurations [5]. Currently, 1145 RBPs have been captured by RNA interactome capture (RIC), of which 595 were discovered to be novel RBPs candidates [6,7]. RNA-binding proteins are strategically located within various cellular compartments to carry out specific functions, ensuring proper cellular functioning. For instance, AtGRDP2 interacts with proteins involved in RNA processing and translation in different compartments: it associates with PABN3 in the nucleus, EF-1α in the cytosol, and CL15 in chloroplasts [8]. While the functional roles of various RBPs in living organisms have been elucidated over the past few decades, it is evident that the functional roles of RBPs in plants still remain significantly underdeveloped [9].

In this review, our focus is directed towards a specific subset of RBPs referred to as glycine-rich RNA-binding proteins (GR-RBPs). These GR-RBPs are typically characterized by the presence of a glycine-rich domain located at their C-terminus. Additionally, they feature either a canonical RNA recognition motif (RRM) or a cold-shock domain (CSD) at their N-terminus. GR-RBPs exhibit notable performance, particularly in response to various stressors, including cold, injury, UV radiation, salinity, and pathogen infections [10,11]. *Arabidopsis thaliana*, as a pivotal tool in the study of plant growth and development, has been subject to extensive investigations concerning the functional roles of AtGRPs. However, it is noteworthy that delving directly into the functions of GR-RBPs in crops holds the potential to significantly bolster support for genetic advancements and agricultural development. In recent years, the identification and study of GR-GRPs have extended to other plant species, such as sweet potato (*Ipomoea batatas*), rice (*Oryza sativa*), maize (*Zea mays*), and tomato (*Solanum lycopersicum*) [11,12,13,14,15,16].

Now, accumulating evidence suggests that GR-RBPs can regulate various aspects of plant physiology, including stomatal movement, seed germination, root, flower, leaf, and fruit growth, through processes such as alternative splicing, RNA editing, or post-transcriptional RNA regulation [17]. Additionally, GR-RBPs play a pivotal role in assisting plants in responding to a wide array of biotic and abiotic environmental stresses, such as chilling damage, high temperatures, drought, salt stress, mechanical damage, and others, through mechanisms like RNA export, phase separation, and polyadenylation [10,18]. In this review, we will delve into the diverse domain characteristics of GR-RBPs and explore several of their novel post-transcriptional regulatory mechanisms in detail. Furthermore, we will systematically summarize the functions of GR-RBPs in various crops, with the aim of providing insights for the application of plant genetic engineering in agricultural research.

## 2. Structural Features of the GR-RBPs

**Figure 1:**
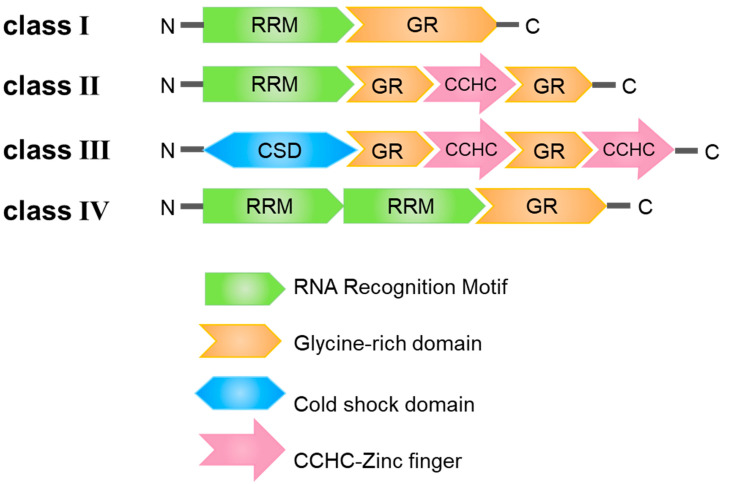
Structural domain characterization of four classes of glycine-rich RNA-binding proteins (GR-RBPs) in plants. RRM: RNA Recognition Motif; GR: Glycine-rich domain; CSD: Cold shock domain; CCHC: CCHC-Zinc finger.

Glycine-rich RNA-binding proteins can be systematically categorized into four discernible classes, denoted as Class I through IV. Class I is characterized by the presence of an RNA recognition motif (RRM) domain in conjunction with a glycine-rich (GR) motif. Class II features a single RRM domain alongside two glycine-rich (GR) motifs, with a Cys3His (CCHC) zinc finger motif positioned between these two glycine-rich domains. Class III stands out due to its unique configuration, where the N-terminal RRM domain is replaced by a cold-shock domain (CSD), followed by two GR-CCHC motifs. Lastly, Class IV is distinguished by the presence of two RRM domains and a GR domain (Figure 1) [17]. These diverse structural domains within GR-RBPs fulfill a spectrum of distinct functional roles.

### 2.1. Glycine-Rich Domain

Glycine-rich proteins were first identified in Petunia in 1986 [19]. They are characterized by glycine motifs consisting of repeating glycine residues, with glycine content ranging from 20% to 70% [17]. Glycine-rich domains are quite prevalent across various organisms, initially leading to the classification of proteins rich in glycine domains into a superfamily [20]. However, some studies have suggested that GRPs may not constitute a distinct protein superfamily, but rather a group of proteins sharing only certain repetitive structural motifs [21]. Within the realm of plants, the diversity in expression patterns, bioregulation, and subcellular localization of such proteins implies that different motif types perform distinct functions. Therefore, multiple GRPs within an organism are likely involved in various physiological and biochemical processes.

GR-RBPs can be distinguished from other GRPs by the presence of nucleic acid binding domains. Furthermore, in addition to glycine, the GR domain also encompasses substantial quantities of aromatic and basic amino acids [22]. The GR domain plays a primary role in mediating interactions between GR-RBPs and other proteins [23]. Meanwhile, the GR domain is an intrinsically disordered region that can drive the onset of phase separation [24]. In Arabidopsis, the glycine-enriched RNA-binding proteins AtRBGD2 and AtRBGD4 can improve the heat tolerance of plants by phase separation [25].

Proteins containing the GR domain have the capacity to induce the formation of discontinuous secondary structures. One is the glycine loop, which forms a nylon clasp-like structure by the interaction between the helices [26]. The other is a structure consisting of β-folds comprising a variable number of anti-parallel chains. The side chains of non-glycine amino acids are oriented toward the same side of the β-fold, resulting in a hydrophobic domain surface that strongly interacts with the hydrophobic regions of its binding partner proteins and macromolecules, thus facilitating the binding of GRP to other proteins [27]. Generally, the GR domain primarily mediates protein–protein interactions. However, it should be noted that the GR domain has also been found to be involved in RNA binding, as seen in tobacco cells where the glycine-rich domain of RZ-1 is implicated in RNA binding [28]. In addition, glycine-rich proteins are structural components of plant cell wall proteins [29]. In rice, OsGRP1 was found to regulate cell elongation of the root [30].

### 2.2. RNA Recognition Motif

The RNA recognition motif (RRM), also known as the RNA-binding domain (RBD) or ribonucleoprotein domain (RNP), represents the most extensively studied RNA-binding protein motif [31]. RRM was first identified in 1988 [32]. An RRM typically comprises approximately 90 amino acid residues, featuring two conserved sequences referred to as RNP1 and RNP2 [32,33]. RNP1 incorporates eight conserved residues, primarily aromatic and positively charged amino acids, denoted as Lys/Arg-Gly-Phe/Tyr-Gly/Ala-Phe/Tyr-Val/Ile/Leu-X-Phe/Tyr, where X can denote any amino acid. In contrast, the sequence of RNP2 exhibits lower conservation compared to RNP1 and consists of a six-residue sequence Ile/Val/Leu-Phe/Tyr-Ile/Val/Leu-X-Asn-Leu located at the N-terminus of the domain. The typical RRM adopts a structural configuration comprising four anti-parallel beta-strands and two alpha-helices, organized into a β1α1β2β3α2β4 sandwich structure. Notably, RNP1 and RNP2 are situated at β3 and β1, respectively [33].

In eukaryotic proteins, RRMs are frequently encountered in multiple copies within a single protein and may coexist with various other domains for binding single-stranded nucleic acids. Notable examples include zinc fingers of the CCCH and CCHC types, the C-terminal domain of polyadenylate-binding proteins (PABP or PABC), and the WW domain [34,35]. Through their interactions with diverse protein domains, the RRM domain can finely tune its RNA-binding affinity and specificity, thereby diversifying its range of biological functions. Mutations occurring within the RRM domain frequently result in a notable reduction or complete abrogation of RNA binding capability [36]. In addition, the affinity of the RRM for RNA binding is influenced by factors such as the N- and C-terminal regions, interdomain linker, and the secondary structure of RNA [37,38]. Moreover, biochemical and structural studies have demonstrated that the RRM not only participates in RNA recognition but also plays a role in mediating protein–protein interactions. The RNA recognition motif (RRM) domain within the protein eIF3b has the capacity to engage with 69 distinct peptides located at the N-terminus of eIF3j. This interaction assumes a pivotal role in the initiation of protein synthesis within eukaryotic cells by eIF3b [39]. In rice, the RRM domain and GR domain of RBP-P are essential for its association with RBP-L and RBP-208 [23].

### 2.3. Cold-Shock Domain

The most prominent distinction among the four categorized groups of GR-RBPs is the unique inclusion of the cold-shock domain (CSD), a feature exclusively found in class III. Consequently, this specific class of GR-RBPs is commonly referred to as CSD proteins (CSDPs) due to the presence of the CSD domain [40]. The capacity of the cold-shock domain (CSD) to enhance the binding of CSD proteins to RNA, single-stranded DNA (ssDNA), and double-stranded DNA (dsDNA) bestows upon them a critical role in the regulation of responses to low temperatures, embryogenesis, flowering time, and fruit development [41].

CSD was first identified in *Escherichia coli* in 1987 [42]. CSD-containing proteins are predominantly observed in bacteria, accounting for 90.9% of occurrences, with 7.3% found in eukaryotes, and plants constituting 0.94% [43]. Eukaryotic CSDs and bacterial CSPs exhibit striking similarity in terms of length, both being approximately 70 amino acids long, and they share conserved sequences, notably RNP1 and RNP2 [44]. In animal and bacterial cells, cold-shock proteins containing CSDs are highly induced in response to cold stress, where they function as RNA chaperone proteins to facilitate efficient translation at low temperatures [45]. In larger eukaryotic proteins, one to five (or even more) CSDs are commonly found, typically associated with naturally unfolded polypeptide regions. A noteworthy observation is that 20% of proteins featuring CSD also possess Zinc Finger CCHC domains, a characteristic shared with class III GR-RBPs. The coexistence of these structural domains may allow CSD-containing proteins to be more diverse in terms of structural domain organization and cellular function [9].

### 2.4. CCHC Motif

The CCHC motif, also referred to as the zinc knuckle, is present in both class II and class III. It represents a small structural motif characterized by multiple finger-like protrusions that establish tandem contacts with their target molecules. Some of these domains have an affinity for binding metal ions such as zinc and iron [46]. Generally speaking, the CCHC motif shares the consensus sequence CX2CX4HX4C (X for any amino acid, numbers for the number of residues, C and H for cysteine and histidine, respectively) [47]. CHC motifs with a high affinity for DNA and RNA typically consist of a short helix and two short β-strands connected through a zinc knuckle structure. These motifs play crucial roles in regulating the function of nucleic acids and participating in transcriptional or translational regulation by binding to target RNA [48].

Proteins containing the CCHC motif are prevalent in plants, often occurring in tandem with other domains, which could be pertinent to specific biological functions [49]. In the context of cold resistance, the presence of both the CSD at the N-terminus and the CCHC motif at the C-terminus, along with the glycine enrichment domain, collectively form the cold-shock domain proteins in plants. In wheat, for instance, out of the 50 CCHC motif-containing proteins, 28 are tandemly arranged with the RRM domain, and 8 are associated with the CSD domain. Promoter analyses of the *TaCCHC-ZFP* genes have revealed the presence of 636 cis-elements responsive to environmental stress and phytohormones. This finding strongly suggests that this domain plays a pivotal role in plant development and stress responses mediated by phytohormones [50]. The count of CCHC motifs varies among cold-shock domain proteins (CSDPs), and the organization of zinc finger domains, both in terms of their number and length, has been documented to play a crucial role in the complete functionality of CSDPs during cold adaptation. In the case of cabbage (*Brassica oleracea*), allelic variation in BoCSDP5 with seven CCHC-type zinc fingers exhibits significantly greater resistance to low temperatures when compared to BoCSDP5 variants containing six CCHC-type zinc fingers [51]. GR-RBPs that incorporate CCHC zinc finger motifs are referred to as zinc finger-containing glycine-rich RNA-binding proteins (RZs). Transcript levels of Arabidopsis AtRZ-1a, AtRZ-1b, and AtRZ-1c were significantly elevated in response to cold stress treatments [13,52]. Furthermore, three members of the RZ family, namely TaRZ1, TaRZ2, and TaRZ3, each harboring CCHC domains, have demonstrated distinctive responsiveness to distinct abiotic stressors [53].

## 3. Roles of GR-RBPs in RNA Metabolism

The presence of the RRM domain endows RNA-binding proteins with involvement in various facets of RNA processing metabolism. These include 5′-capping, alternative splicing, 3′ polyadenylation, RNA stability, and translation. Ultimately, these RNA-binding proteins play a pivotal role in regulating gene expression as well as plant growth and development [54]. Below, we provide a detailed description of several RNA processing functions mediated by glycine-rich RNA-binding proteins, emphasizing their significance in plant regulation.

### 3.1. RNA Alternative Splice and Polyadenylation

Polyadenylation of the 3′-terminus of precursor mRNA plays an important role in eukaryotic gene expression and regulation. This process comprises two closely linked biochemical steps: the initial cleavage at the poly(A) sites (PAS) of the precursor mRNA, followed by the addition of the poly(A) tail [55]. The selection of PAS is variable, a phenomenon known as alternative polyadenylation (APA), which represents a widespread mechanism for regulating gene expression across various species [56]. APA adds complexity to the transcriptome and can regulate the function, stability, localization, and translation efficiency of target RNAs [56].

**Figure 2:**
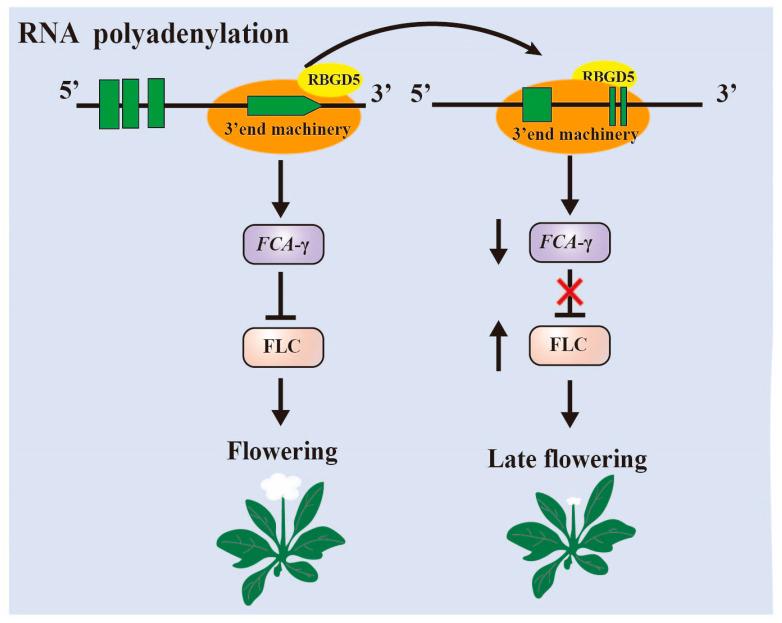
RBGD5 affects the selection of polyadenylation sites at the 3′ end of Arabidopsis FCA precursor mRNAs. The shift of the Poly(A) site from distal to proximal when RBGD5 is mutated results in upregulation of FLC and delayed flowering.

AtRBGD5, also known as HLP1, plays a pivotal role in the selection of polyadenylation sites at the 3′ end of precursor mRNAs in Arabidopsis (Figure 2) [36]. HLP1 is notably enriched in transcripts associated with RNA metabolism and flowering processes. The homozygous deletion mutant of HLP1, known as hlp1-1, exhibits a late flowering phenotype. CLIP-seq analysis has revealed that HLP1 exhibits a preferential binding affinity for A- and U-rich motifs located in proximity to cleavage and polyadenylation sites. A comprehensive examination of poly(A) site usage has shown that HLP1 deletion mutations lead to the translocation of thousands of poly(A) sites. Notably, the flowering regulator FCA is identified as a direct target of HLP1. HLP1 actively promotes polyadenylation at the distal site of FCA. Consequently, when HLP1 is mutated, there is a shift of the poly(A) site (PSA) from distal to proximal, resulting in the upregulation of FLC and a delay in flowering.

Notably, AtGRP7 has also been documented to bind to antisense precursor mRNAs of FLC and to associate closely with the polyadenylation site (PAS) of the proximal COOLAIR structural type. The loss of AtGRP7 function results in a decrease in the proximal-distal polyadenylation ratio and an increase in the overall abundance of FLC transcripts, ultimately leading to a delay in flowering time [57]. Moreover, AtGRP7 is the first glycine-rich protein in plants known to regulate alternative splicing through direct binding to pre-mRNAs [58]. Furthermore, it has been reported that two functionally redundant RNA-binding proteins, RZ-1B and RZ-1C (collectively referred to as RZ-1) in Arabidopsis, play a vital role in regulating RNA splicing. The loss of RZ-1 function leads to pleiotropic phenotypes, including delayed seed germination, accompanied by the defective splicing of numerous genes and a global perturbation of gene expression. AtRZ-1c, specifically, can bind to FLC, thereby promoting the efficient splicing of FLC introns and repressing FLC transcription, which in turn affects flowering time in Arabidopsis [59].

### 3.2. miRNA Biogenesis

Post-transcriptional regulation holds a significant role in coordinating gene expression in eukaryotes. Among the crucial players in this RNA-level control are microRNAs (miRNAs). In plants, the significance of miRNAs is underscored by the occurrence of severe developmental defects in mutants with impaired miRNA biogenesis. miRNAs are generated through the endonucleolytic cleavage of long primary microRNAs (pri-miRNAs) featuring internal stem-loop structures. RNA binding proteins (RBPs) play a specific role by interacting with well-defined sequence motifs, thereby exerting control over the generation of miRNAs [5,60].

**Figure 3:**
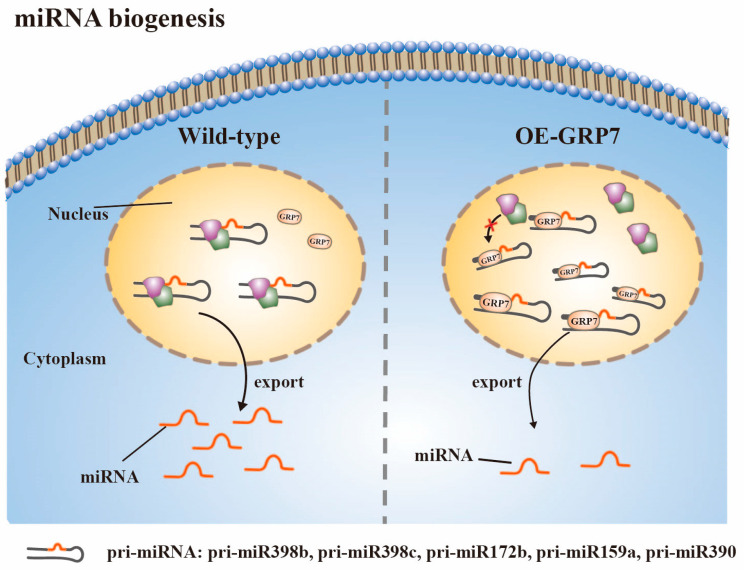
AtGRP7 directly binds pri-miRNAs (pri-miR398b, pri-miR398c, pri-miR172b, pri-miR159a, pri-miR390) in vivo, affecting their processing. Overexpression of AtGRP7 results in an increase in some pri-miRNAs and a decrease in their corresponding mature miRNAs.

It has been observed that AtGRP7 exerts an impact on the inventory of miRNAs (Figure 3). The overexpression of AtGRP7 resulted in a significant decrease in the levels of 30 miRNAs and an increase in the levels of 14 miRNAs. Simultaneously, there is an over-accumulation of certain pri-miRNAs, including pri-miR398b, pri-miR398c, pri-miR172b, pri-miR159a, and pri-miR390 upon the upregulation of AtGRP7. Interestingly, the expression levels of their corresponding mature miRNAs are decreased. RNA immunoprecipitation experiments have demonstrated that AtGRP7 directly binds to these pri-miRNAs in vivo, thereby affecting their processing and inhibiting the synthesis of the corresponding mature miRNAs [61]. More recently, researchers have also shown that AtRZ1A is involved in miR398 biogenesis. In atrz-1a mutants, there is a decrease in the steady-state levels of mature miR398, along with a decrease in pri-miR398b levels [62]. This decrease may be attributed to the fact that AtRZ-1a appears to specifically influence the stability of pri-miR398b. Interestingly, these findings contrast with the results observed with AtGRP7, suggesting that GR-RBPs regulate miRNA biogenesis through different mechanisms.

The interaction between miRNAs and RBPs offers a broader perspective for studying the mechanisms underlying growth, development, and stress responses in plants. There is still much to uncover about the synthesis, transport, and degradation of GR-RBPs and miRNAs, and these aspects promise to unveil further insights into the intricate processes governing plant biology.

### 3.3. RNA Assembly by Liquid–Liquid Phase Separation

In cell biology, the concept of liquid–liquid phase separation (LLPS) refers to a state in which biomolecules become spatially concentrated within the cell, leading to the formation of various membraneless cytoplasmic and nuclear compartments. There is a growing body of evidence indicating that LLPS is closely intertwined with several fundamental cellular processes, serving a crucial role in the regulation of gene expression, cell division, signal transduction, stress responses, cytoskeletal dynamics, supramolecular assembly, and more [63]. In plants, LLPS has been reported to play a role in sensing high temperatures, water potential, phytohormones, and responses to pathogens [64]. Currently, significant breakthroughs are being achieved in the functional studies of phase separation in plants, shedding light on its intricate mechanisms [65].

In 2018, it was discovered that AtGRP7 interacts with receptor kinase FERONIA (FER) and undergoes phosphorylation by FER at six serine/tyrosine residues, which enhances the GRP7 protein accumulation in the nucleus and increases the ability of GRP7 to bind its target mRNAs [66]. The FER-dependent phosphorylation of GRP7 enhances its association with the spliceosome component U1-70K, facilitating splice site selection and thereby modulating dynamic alternative splicing (AS). In response to changing external environmental conditions, AtGRP7 acts through the Rapid Alkalinization Factor 1 (RALF1)-FERONIA signaling pathway. It regulates the alternative splicing of its target genes, allowing for adjustments to the transcriptome in response to various stresses in plants. This mechanism highlights the role of AtGRP7 in fine-tuning gene expression in plants under changing environmental conditions.

**Figure 4:**
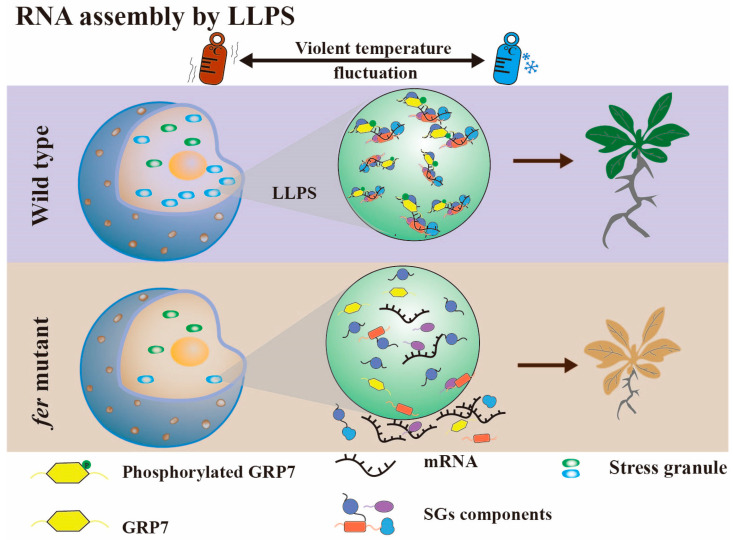
When the temperature changes violently between hot (red thermometer) and cold (blue thermometer), phosphorylated GRP7 in the cytoplasm can be used as a scaffold protein to recruit RNA and other proteins to form SGs by liquid–liquid phase separation, which facilitates RNA assembly and subsequently blocks translation to ensure normal root elongation.

Recent findings have revealed that FER plays a pivotal role in mediating the liquid–liquid phase separation (LLPS) of GRP7 in response to temperature fluctuations in Arabidopsis (Figure 4) [67]. Under conditions of temperature shifts, GRP7 in the cytoplasm serves as a scaffold protein, recruiting RNA and other molecules, including the RNA chaperones CSP1 and CSP3, as well as the translation-related protein eIF4E. This assembly of molecules undergoes LLPS, leading to the formation of stress granules (SGs). These SGs facilitate the assembly and sequestration of RNA, ultimately blocking translation, thereby ensuring normal root elongation. Conversely, the grp7 mutant was sensitive to fluctuating temperature change, with poor growth and shortened primary root length under the same conditions. Additionally, FER-dependent phosphorylation is crucial for regulating GRP7 phase separation. In fer mutant plants, the LLPS ability of GRP7 is diminished, leading to a phenotype similar to that of the grp7 mutant, which in turn hampers root growth. It is noteworthy that GRP7 in the nucleus also undergoes phase separation, along with a component of the spliceosome, U1-70K. This suggests that phase separation may also be involved in RNA alternative splicing processes.

### 3.4. Translation of RNA

Translation is a fundamental process within the genetic central dogma of molecular biology, playing a crucial role in ensuring the accurate execution of RNA functions. At present, the GR-RBPs that are directly involved in the translation of RNA targets remain largely unknown.

**Figure 5:**
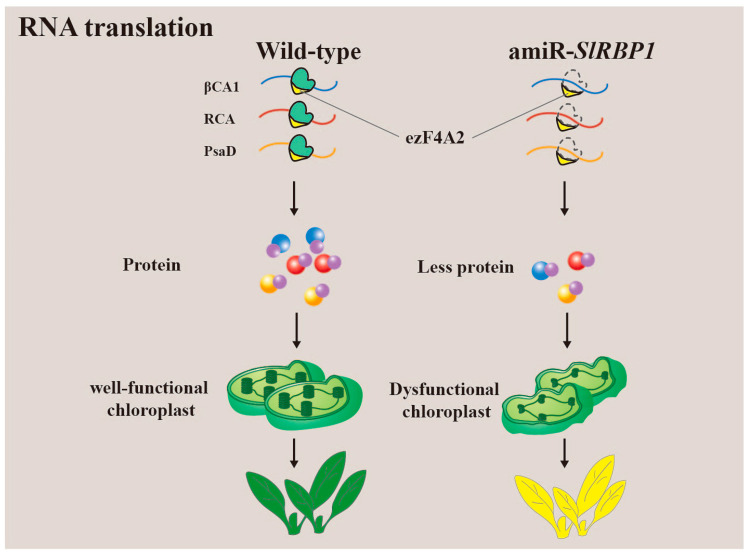
SlRBP1 interacts with SleIF4A2 to together maintain chloroplast function by ensuring translation of photosynthesis-associated transcripts. Loss-of-function of SlRBP1 results in impaired chloroplast ultrastructure, downregulated photosynthesis rate, and dwarf tomato plants with yellow leaves.

Recent research has highlighted the role of a glycine-rich RNA-binding protein (GR-RBPs), SlRBP1, in the maintenance of chloroplast function in tomato (Figure 5) [68]. SlRBP1 exhibits specific binding to genes associated with photosynthesis and chloroplast function, including β carbonic anhydrase 1 (βCA1), Rubisco activase (RCA), and photosystem I reaction centre subunit II (PsaD). Furthermore, SlRBP1 interacts with SleIF4A2, and their collaboration serves to uphold chloroplast function by enhancing the translatability of transcripts associated with photosynthesis. In cases where SlRBP1 is non-functional, the transcription levels of its target genes remain unchanged, but translation is significantly impaired. Consequently, this leads to structural abnormalities in chloroplasts, a decreased rate of photosynthesis, and the development of dwarf tomato plants with yellow leaves. This research underscores the critical role of SlRBP1 in regulating chloroplast function, and, by extension, plant growth and development.

## 4. Roles of GR-RBPs in Crops

Currently, GR-RBPs have been identified as key players in the regulation of a diverse array of physiological processes in plants [10]. These encompass critical aspects such as leaf development, root elongation, and various facets of vegetative growth. Furthermore, GR-RBPs exert substantial influence over reproductive phenomena including flowering and fruit development. Notably, they also assume a pivotal role in bolstering plant resilience against an array of stressors, spanning cold, heat, salinity, drought, and biotic stress. To foster a deeper comprehension of GR-RBPs and to broaden their pragmatic utility in the realm of agricultural production, this synthesis endeavors to consolidate and elucidate the multifaceted functions of GR-RBPs within the context of diverse crop species.

### 4.1. GR-RBPs in Grain Crops

Grain crops are the main sources of human food, which are mainly divided into cereal, potato, and legume crops. Grain crops have been cultivated for an extended period due to their lower water content and increased resistance to storage. Presently, three of these crops, namely wheat, rice, and maize, collectively contribute to more than half of the world’s food supply. The role of GR-RBPs has also been investigated in these important crop species.

#### 4.1.1. GR-RBPs in Rice

In the case of rice, glycine-rich RNA-binding proteins have been identified as responders to various abiotic stress conditions, including soil salinity, suboptimal temperatures, and varying degrees of drought [69]. Among the six glycine-rich RNA-binding proteins (OsGRP1 to OsGRP6) in rice, exposure to cold stress significantly upregulated the expression of all six genes [12]. Notably, OsGRP1 and OsGRP6 were capable of restoring cold acclimation in cold-sensitive E. coli mutant strains. Furthermore, OsGRP1 and OsGRP4 played roles in promoting seed germination and seedling growth under low-temperature conditions. Additionally, OsGRP4 and OsGRP6 facilitated mRNA export from the grp7 mutant during cold stress, aiding in nuclear-to-cytoplasmic export. Among the three zinc finger-containing GR-RBPs in rice (OsRZ-1, OsRZ-2, and OsRZ-3), OsRZ-2 demonstrated the ability to rescue cold sensitivity in grp7 mutants, affirming the functional conservation of these proteins in responding to cold stress [70].

In recent years, OsGRP3 has emerged as a key positive regulator of drought tolerance in rice. Rice plants overexpressing OsGRP3 exhibited improved growth and higher survival rates following drought treatment. Conversely, transgenic rice plants with OsGRP3 knocked out or knocked down displayed reduced survival rates under drought conditions. Notably, Jae et al. found that OsGRP3 plays a role in modulating the transcript levels and mRNA stability of ROS-related genes, such as Peroxidase 1 (POD1) and LIPOXYGENASE (LOX), thereby regulating ROS accumulation in response to drought stress. This regulatory mechanism contributes to enhanced drought tolerance [69]. Furthermore, Xu et al. demonstrated that several peroxidase (POD) enzymes involved in lignin biosynthesis were downregulated in the Osgrp3 mutant line. OsGRP3 was found to enhance drought tolerance in rice by influencing the phenylpropanoid biosynthesis pathway and subsequently modulating lignin accumulation. These findings underscore the multifaceted role of OsGRP3 in enhancing rice’s resilience to drought stress [71]. In summary, OsGRP3 plays a pivotal role in enhancing drought resistance in rice by positively regulating lignin biosynthesis and promoting the scavenging of H_2_O_2_ through peroxidases (PODs). Additionally, the expression levels of OsGRP2 and OsGRP4 were found to increase in response to drought treatment, while the expression levels of OsGRP1, OsGRP5, and OsGRP6 decreased. These observations suggest that these glycine-rich RNA-binding proteins (GRPs) may also contribute to drought resistance in rice [69]. Furthermore, OsGRP4 was identified in a rice heat shock cDNA library. The exogenous overexpression of OsGRP4 in yeast cells resulted in increased thermotolerance, highlighting its potential role in enhancing heat tolerance as well [72].

OsGRP1 was found to promote cell expansion as a post-transcriptional regulator downstream of the Brassinosteroids (BR) signaling pathway. Overexpression of OsGRP1 in Arabidopsis inhibited the dwarf phenotype of the BR-insensitive mutant bri1-5 [73]. Indeed, the glycine-rich protein family is a branch of plant cell wall proteins. OsGRP1 was found to be present in the cell elongation and differentiation regions of root and young tissues in as early as 1995 [30]. OsGRP2 also exhibited vascular-specific expression and played an important role in the construction of the cell wall network in the early development of the floral organ [74]. OsGRP7 were identified as being highly expressed in root with no expression in other organs in rice. A GFP reporter gene driven by the OsGRP7 gene promoter showed a strong root-specific expression pattern and stable expression, which provided a useful technique for the genetic manipulation of wheat root traits [75]. However, the evidence for the involvement of GRPs in cell elongation or expansion to regulate plant development requires further study.

Moreover, the glycine-enrich RNA-binding protein in rice endosperm, OsRBP-P, can interact with itself, OsRBP-L, and OsRBP-208. OsRBP-P and OsRBP-L are responsible for the zipcode binding of glutenin and glutamolysin mRNAs in endosperm cells, allowing their correct localization. Mutations in either OsRBP-P or OsRBP-L result in the mis-localization of glutenin and glutamolysin mRNAs, disrupting the transport of their corresponding proteins in rice endosperm. Both rbp-l or rbp-p knockdown mutants exhibited plant dwarfism, chlorophyll deficiency, sterility to late flowering, and low spikelet fertility [23,76]. Interestingly, OsGRP3 was localized in the nucleus and cytoplasm. OsGRP3-GFP was transported from the cytoplasm/nucleus into cytoplasmic foci following exposure to ABA and mannitol treatments [71]. In rice leaf cells and rice protoplasts, OsGRP4 was shown to be localized in the nucleus. After 42 °C heat shock, OsGRP4 shuttled from the nucleus to the cytoplasm [72]. These results suggest that OsGRPs may respond to different environmental stresses by regulating the transport of stress-related genes.

#### 4.1.2. GR-RBPs in Wheat

whGRP-1 in wheat was the first to be cloned back in 1996. Its expression is notably high in the roots and low in the leaves of wheat seedlings. Interestingly, the expression of whGRP-1 in wheat endosperm significantly increased after exposure to exogenous ABA treatment. This suggests that this protein may have a role in the response of wheat to drought stress [77]. Three members of the RZ family, namely TaRZ1, TaRZ2, and TaRZ3, have been identified as responsive to various abiotic stresses. Overexpressing TaRZ1 in Arabidopsis led to delayed seed germination and suppressed seedling growth under conditions of salt stress, as well as inhibited seedling growth at low temperatures. Transgenic plants overexpressing TaRZ2 or TaRZ3 also exhibited delayed seed germination in response to salt stress and drought conditions. However, the overexpression of TaRZ2 conferred greater freezing tolerance to the transgenic plants [53].

TaGRP2 also plays a significant role in regulating wheat flowering as a flowering repressor. The overexpression of TaGRP2 resulted in delayed flowering, while TaGRP2-RNAi plants exhibited accelerated flowering compared to the wild type. Furthermore, TaGRP2 undergoes O-GlcNAcylation modification, which is involved in regulating flowering in response to prolonged cold during the vernalization of winter wheat. TaGRP2 functions by inhibiting the accumulation of transcripts of the key flowering-promoting gene, TaVRN1, through direct binding to a critical region within the first intron of TaVRN1 pre-mRNA [78]. During vernalization, O-GlcNAc modification of TaGRP2 within the nucleus gradually increases. Nuclear-localized VER2 interacts with O-GlcNAc-modified TaGRP2, potentially leading to its reduced accumulation in the nucleus and/or attenuating its binding to TaVRN1. This release of repression on TaVRN1 ultimately promotes flowering.

#### 4.1.3. GR-RBPs in Maize

MA16 was the first identified glycine-rich RNA-binding protein in maize. It was found to be induced by abscisic acid in immature embryos [79] and has been linked to various environmental stresses, including drought, wounding, and heavy metal treatment [80]. MA16 exhibits a wide range of RNA-binding activities, with a particularly high affinity for ribosomal RNA (rRNA) [81]. Imunoelectron microscopy studies have revealed that MA16 is localized in the cytoplasm, nucleus, and dense fibrillar components of the nucleolus, where it plays a role in rRNA processing [82]. Moreover, MA16 has been shown to interact with a DEAD box RNA helicase called ZmDRH1, which is also located in the nucleus and nucleolus and binds to fibrillarin. These findings suggest that MA16 may participate in rRNA metabolism by forming a ribonucleoprotein complex with ZmDRH1 and fibrillarin [83].

The upregulation of ZmGRP2 appears to enhance maize’s resistance to Meloidogyne arenaria infection, potentially due to its cell wall-associated functions [84]. Similarly, ZmGRBP2 was found to be highly expressed specifically in Aspergillus flavus-susceptible maize lines, Va35, indicating that it affects maize host hypersensitivity and susceptibility to A. flavus [85]. Moreover, glycine-rich RNA-binding protein 1(ZmGRP1) plays roles in the splicing and nuclear export of photoperiodic mRNAs, thereby controlling the RNA response to changes in photoperiod in maize [86].

A total of 23 ZmRB-GRP genes from four classes have been identified in maize to date. Except for ZmGRP11 and ZmGRP16, most of the ZmRB-GRPs exhibit high expression levels in all tissues, suggesting that they play important roles throughout the maize life cycle. Among these genes, ZmRB-GRP5 shows the highest expression levels in all tissues. Predictions of cis-elements in the promoter sequences of these ZmRB-GRPs revealed three classes of elements: ABA-responsive elements (ABREs), drought-responsive elements (DREs), and cold-responsive elements (CREs). Eight ZmGRPs from four subclasses were selected for stress experiments involving cold, salt, abscisic acid (ABA), and recovery from chilling, and it was observed that they significantly responded to two to four of these stress conditions [13]. This suggests that ZmRB-GRPs can regulate their own expression in response to environmental stress.

#### 4.1.4. GR-RBPs in Other Crops

Potato crops mainly include sweet potatoes, potatoes, yams, etc., with sweet potatoes and potatoes being the main food crops suitable for various purposes such as food, vegetables, animal feed, and industrial raw materials. In 2019, the nine ItGRPs in sweet potatoes (*Ipomoea trifida*) were characterized. Among these, ItGRP9 was highly expressed in flowers, while the remaining ItGRPs showed significant expression in young leaves. However, they exhibited diverse expression patterns across different tissues of sweet potatoes. Additionally, these genes displayed differential regulation in response to various stresses like cold, heat, drought, and salt stresses [16]. For instance, the expressions of ItGRP1, ItGRP5, and ItGRP7 increased with rising temperatures and under heat stress, suggesting their involvement in sweet potato growth and development. It is worth noting that there is limited research on GR-RBPs in potato crops. Currently, only St-mRBP2 has been identified, which is localized in mitochondria and possibly plays a role in the regulation of mitochondrial gene expression [87]. Therefore, further research on GR-RBPs in tuber crops like potatoes is needed.

In plants, there are cases of persistent plant virus infections that are transmitted exclusively through vertical means, such as through seeds, and do not exhibit obvious symptoms. This phenomenon has been observed in the context of plant mitotic viruses. In quinoa crops infected with mitotic viruses, the levels of the CqGR-RBP2 protein were significantly increased. Interestingly, quinoa plants carrying mitoviruses demonstrated improved drought resistance. CqGR-RBP2 is believed to be involved in this process, as the overexpression of CqGR-RBP2 in Arabidopsis enhanced drought resistance. This discovery opens up a new perspective for the genomic breeding of crops and warrants further investigation and research [88].

Additionally, in barley, HvGR-RBP1 has been found to play a role in the regulation of flowering and senescence processes. Furthermore, HvGR-RBP1 exhibits RNA folding chaperone activity, akin to its homolog AtGRP7. When subjected to cold treatment, the transcript levels of HvGR-RBP1 increase. In E. coli cells expressing HvGR-RBP1 heterologously, these cells exhibited faster growth compared to control cells following exposure to cold shock. It is worth noting that the RRM structural domain of HvGR-RBP1 is crucial for this function [89]. And, also, the mRNA level of HvGRP3 was significantly increased under cold treatment and fungal pathogen Erysiphe graminis and Rhynchosporium secalis infestation [90]. Noticeable increases in the expression of SbGR-RBP were observed in sorghum under various concentrations of salt treatment [91].

In summary, GR-RBPs have been extensively investigated in grain crops such as maize, rice, wheat, barley, and potato. They have been shown to play roles in both biotic and abiotic stress responses, and also influence plant flowering and cell wall composition (Table 1).

### 4.2. GR-RBPs in Horticultural Crops

Horticultural crops typically refer to the cultivation of crops on a smaller scale and are broadly categorized into three main groups: fruit trees, vegetables, and ornamental plants. In today’s world, horticultural products have become essential for enhancing human nutrition, as well as for beautifying and purifying the living environment.

#### 4.2.1. GR-RBPs in Fruits

Tomato is the largest vegetable crop in the world. Recently, an RNA-binding protein, SlRBP1, which maintains chloroplast function by regulating the translation of bound target RNAs has also been identified in tomato. The deletion of SlRBP1 resulted in dwarf growth and the yellowing of leaves. In addition, miRNA silencing of SlRBP1 resulted in significantly smaller tomato fruits [68]. However, the exact regulatory mechanism of this phenomenon requires further study. This suggests that GR-RBPs may also be involved in the regulation of fruit size. Since tomato is a model plant for studying the molecular mechanisms of fruit development and ripening due to its short life cycle, high reproduction rate, and self-pollination, among other factors [92], GR-RBPs in tomato have been proven to play a role in fruit ripening.

It was reported that the SlORRM4 (glycine-rich RNA-binding protein 5), which functions as a major mitochondrial editing factor, is involved in fruit ripening in tomato [93]. The fruit ripening start time in Slorrm4 was significantly delayed compared to the wild type (6–8 days), accompanied by the delay and decrease in respiratory and ethylene peak, ultimately resulting in the fruit failing to turn completely red. This phenomenon is attributed to the compromised RNA editing of genes associated with mitochondria, such as nad3 and sdh4. This impairment interferes with the proper assembly of mitochondrial respiratory complexes, resulting in a disruption of mitochondrial structure and function. Consequently, this disruption hampers the fruit’s respiration rate, ultimately culminating in a delay in the initiation of fruit ripening [94].

Meanwhile, RZ1A-Like (RZ1AL), a member of the RZ protein family, plays an important role in tomato ripening, especially in fruit coloring [95]. As the fruit matured, compared with the wild type, the fruit of the rz1al mutant continued to show an orange color. The content of lycopene in the fruit was significantly reduced, and the number of molecular structures containing carotenoids in the outer epidermis of rz1al mutant fruit decreased significantly. Transcriptomic data showed that RZ1AL regulates the expression of PSY, ZDS, CRTR-B1, and CRTR-B2, key genes of the carotenoid biosynthesis and metabolic pathways. Proteomic analysis showed that a large number of ribosomal proteins were significantly downregulated in rz1al mutant, especially the 60S subunit, and both transcript and protein levels of ZDS were reduced in rz1al mutant fruits. Taken together, this suggests that RZ1AL affects the ripening process of tomato fruits both in terms of mRNA transcription and protein translation. These pieces of evidence demonstrate that GR-RBPs further improve the gene regulatory network of tomato ripening and development.

Considering the function of GR-RBPs in coping with stress in other crops, it is reasonable to believe that they will also help tomato to better face a variety of stress responses. The LeRBP1 in tomato was overexpressed in transgenic fruit using the polygalacturonase promoter, which drives expression in fruits from the mature green stage [96]. Under postharvest cold-storage conditions (7 °C for 4 days), the total protein content of transgenic fruit overexpressing LeRBP1 was significantly higher than that of wild type (WT) fruit, and there were changes in the content of some free amino acids. This suggests that LeGRP1 may play a role in rearranging its structure and/or stabilizing mRNA, allowing for effective translation at low temperatures. The functions of GR-RBPs indeed warrant further in-depth exploration.

Lately, nineteen VviGRPs were identified from the grape genome, and seventeen of them were found to be expressed in ovules [97]. Expression analyses conducted during ovule development revealed distinct expression profiles for most ovule-expressed VviGRPs between seeded and stenospermocarpic grapes. Specifically, genes like VviGRP2, VviRZ-1A, VviGRP3, VviGRP5La, and VviGRP7 displayed significant differences in expression. These findings imply that these five genes may play essential roles in ovule development in stenospermocarpic grape varieties. This indicates that GR-RBPs could offer valuable insights for seedless grape selection and breeding efforts.

#### 4.2.2. GR-RBPs in Vegetables

Cucumber is a chilling-sensitive fruit, and when stored below 7–10 °C, it can develop symptoms of chilling damage, including surface depression, browning, or rotting. These issues significantly affect the quality and storage period of the fruit. To mitigate these problems, plants undergo a process called cold acclimation, which helps them to develop tolerance to cold temperatures after initial exposure to critically low temperatures. This adaptation process is effective in reducing chilling damage in cucumber fruits. In cucumber, there are six *GR-RBPs*, and their expression patterns vary in response to temperature changes during fruit storage. When cucumber fruits were directly stored at 5 °C after harvest, only one of these genes, *CsGR-RBP2*, showed a significant increase in expression. However, the expression of the other five genes, namely *CsGR-RBP3*, *CsGR-RBP4*, *CsGR-RBP5*, *CsGR-RBP-blt801*, and *CsGR-RBP-RZ1A*, showed a decreasing trend. Interestingly, when cucumber fruits were first treated at 10 °C for three days and then stored at 5 °C, all six genes were significantly upregulated. Among them, *CsGR-RBP3* displayed the most significant difference in transcript level expression. Furthermore, Arabidopsis plants overexpressing *CsGR-RBP3* exhibited a faster growth rate, longer primary root length, and more rosette leaves than the wild type at room temperature (23 °C); lower electrolyte permeability, reactive oxygen species levels, and higher catalase and superoxide dismutase activities at 0 °C; and higher survival at −20 °C. These findings suggest that *CsGR-RBP3* has the potential to enhance chilling and freezing tolerance in plants like Arabidopsis [98]. In addition, tobacco transient expression demonstrated that CsGR-RBP3 was localized to mitochondria, indicating that *CsGR-RBP3* may play a role in maintaining mitochondria-related functions at low temperatures.

In oilseed rape, seven *BnGRPs* were characterized. The expression of all *BnGRPs* was upregulated under cold stress, whereas the transcript levels of all *BnGRPs* were downregulated under dehydration or high salt stress. *BnGRP1* successfully complemented the cold-sensitive phenotype of the *Escherichia coli* mutant strain BX04 under cold stress. The expression of *BnGRP1* in Arabidopsis accelerated seed germination and enhanced the cold tolerance of plant growth under cold or freezing conditions [99]. In Chinese cabbage, two *BrGRP* genes, w546 (Bra030284) and w1409 (Bra014000), were also upregulated under salt stress [100].

#### 4.2.3. GR-RBPs in Ornamental Plant

*MpGR-RBP1*, a GRP in *Malus prunifolia*, was found to be upregulated by salt stress, oxidative stress, and ABA treatment. Arabidopsis plants overexpressing *MpGR-RBP1* showed accelerated seed germination and seedling growth under high salt or oxidative stress [101]. Furthermore, electrolyte permeability, malondialdehyde, and ROS accumulation were reduced in transgenic plants under high salt stress, suggesting that this gene contributes to salt tolerance in plants. Additional1y, *MhGR-RBP1* was highly expressed in leaves compared to shoots and roots. Expression levels of *MhGR-RBP1* were elevated under drought, high salt, hydrogen peroxide, and injury stresses [102].

In horticultural crops, GR-RBPs regulates fruit development, ripening, and postharvest storage, and helps plants to better cope with environmental stress. However, there are still few studies on ornamental plants (Table 2).

### 4.3. GR-RBPs in Other Crops

Apart from grain crops and horticultural crops, oil crops, sugar crops, medicinal crops, feed crops, and so on are also integral parts of human production and life. The function of GR-RBPs has also been found in several crops.

Tobacco is an important economic crop in which GR-RBPs have been found to be related to resistance to biological stress. NbGRP7 has been verified to interact with the plant nucleotide-binding leucine-rich repeat (NB-LRR) proteins Gpa2 and Rx1 in *N. benthamiana*, thereby participating in plant effector-triggered immunity. Transient overexpression and silencing assays showed that *NbGRP*7 plays a positive role in Gpa2-mediated cell death and Rx1-mediated extreme resistance. Meanwhile, NbGRP7, as a cofactor regulating the stability of the NB-LRR receptors, can affect the transcript level and protein abundance of the Rx1 receptor through its RNA-binding motif (RRM). However, the exact regulatory mechanism is undefined [103].

Currently, the literature reports an interaction between NbRZ-1A and the soybean blast effector PsFYVE1. This interaction has been shown to regulate not only the pre-mRNA selective splicing (AS) of *NbNSL1* but also the transcription of genes associated with plant immunity, thereby influencing plant immunity against pathogens [104]. In addition, to counteract host defense, many plant viruses encode viral suppressors of RNA silencing targeting various stages of RNA silencing. Conversely, plants also encode endogenous suppressors of RNA silencing (ESRs) that function in the ongoing defense–counterdefense arms race between host plants and viruses. NgRBP, a glycine-rich RNA-binding protein from *Nicotiana glutinosa*, serves as an ESR and suppresses both local and systemic RNA silencing induced by sense- or double-stranded RNA, preventing silencing from spreading systemically. *NgRBP* promotes *Potato Virus X* (PVX) infection in N. benthamiana, while *NgRBP* knockdown enhances PVX and *Cucumber mosaic virus* (CMV) resistance in *N. glutinosa* [105]. Furthermore, when tobacco was subjected to high temperatures, the expressions of NtGRP-1a and NtGRP-3 significantly increased, whereas the expression of NtGRP-1b remained unaffected [15].

*Camelina sativa* L. is an oilseed crop with potential applications in biofuel production. In 2013, Kwak et al. isolated and characterized three GRPs from camelina: *CsGRP2a*, *CsGRP2b*, and *CsGRP2c*. The expression of all three *CsGRP2s* was significantly upregulated under low-temperature stress. *CsGRP2a* showed increased expression levels under salt or dehydration stress, whereas the transcript levels of *CsGRP2b* and *CsGRP2c* decreased under salt or dehydration stress. These *CsGRP2s* demonstrated the ability to complement cold-sensitive *E. coli* mutants at low temperatures [14]. Subsequently, in 2016, three more GRPs were discovered: *CsGRP7a*, *CsGRP7b*, and *CsGRP7c*. The *CsGRP7* genes exhibited universal expression across all plant tissues. Cold stress significantly upregulated the expression of *CsGRP7* genes, while salt stress or dehydration stress had a limited impact on their expression. Overexpression of *CsGRP7a* in Arabidopsis and Camellia plants revealed that *CsGRP7a* positively influenced cold tolerance but negatively affected tolerance to salt or drought stress [106]. Additionally, all *CsGRP2* and *CsGRP7* genes exhibited RNA chaperone activity.

Wang et al. characterized a GRP gene (*LbGRP1*) from *Limonium bicolor*, a medicinal plant. Transgenic plants overexpressing *LbGRP1* exhibited higher peroxide dismutase and catalase activities, as well as higher proline content, under salt-stress conditions compared to wild type tobacco [107]. These results suggest that the overexpression of the *LbGRP1* gene can influence certain physiological processes to enhance salt tolerance in plants. Additionally, *Suaeda asparagoides* is a saline plant with significant food and medicinal value. Transgenic *Arabidopsis thaliana* plants overexpressing *SaRBP1* demonstrated salt tolerance, as evidenced by longer primary roots, increased fresh weight, a higher number of lateral roots, and significantly improved survival rates compared to the wild type under salt-stressed conditions [108]. In addition, Nomata et al. isolated three GRPs in *Physcomitrella patens* named, respectively, *PpGRP1*, *PpGRP2*, and *PpGRP3*. The expression levels of *PpGRPs* transcripts and proteins increased following cold treatment. Notably, PpGRP1 and PpGRP2 were found to be localized in the nucleus, while PpGRP3 was observed in the mitochondria. This localization suggests that PpGRP3 may play a role in the processing of mitochondria-associated RNAs [109]. *Zoysia grass* is an effective sand-fixing plant. The overexpression of the glycine-rich RNA-binding protein ZjGRP in knotweed enhanced Arabidopsis’ resistance to salt stress, indicating the potential application of GR-RBPs in environmental improvement [110].

GR-RBPs in tobacco has been found to be related to plant immunity and can improve its resistance to viruses. In other reported species, GR-RBPs are also associated with cold stress and salt stress (Table 3).

## 5. Conclusions

**Figure 6:**
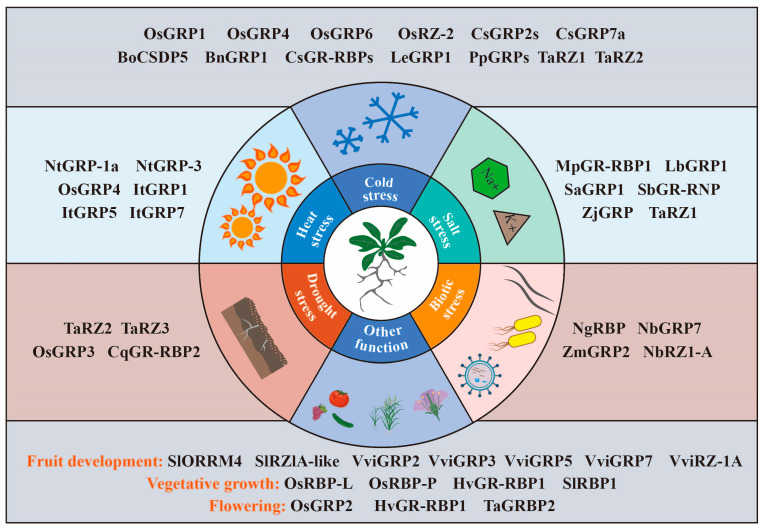
Overview of the functional roles of GR-RBPs in crop plants. GR-RBPs in crops can not only help crops better resist stress responses (cold, heat, salt, drought, virus, and pest infection), but also regulate plant development, such as vegetative growth, flowering time, and fruit development.

Glycine-enrich RNA-binding proteins (GR-RBPs) are central players in post-transcriptional regulation, and their functions have been extensively studied in the model plant Arabidopsis thaliana [8,45,111,112]. GR-RBPs regulate gene expression and cell fate through various RNA processing mechanisms, including alternative splicing, polyadenylation, editing, as well as RNA transport, stabilization, translation, and degradation. However, a comprehensive summary of the functions of GR-RBPs in other crops is lacking. In this review, we provide a detailed overview of the domain characteristics of GR-RBPs and explore several newly discovered regulatory mechanisms, such as liquid–liquid phase separation (LLPS) and RNA translation (Figure 2, Figure 3, Figure 4 and Figure 5) [25,67]. Furthermore, we have compiled information on the functions of GR-RBPs in all reported crops, excluding *Arabidopsis thaliana*, to offer a comprehensive perspective (Figure 6).

In comparison, the functions of GR-RBPs in crops primarily revolve around responding to various stress conditions, with cold stress response genes being the most commonly reported. The overexpression of *GR-RBPs* often enhances the freezing resistance of crops, such as *CsGR-RBP3* and *LeGRP1*, although there are exceptions, such as *TzRZ1*. In general, GR-RBPs do not exclusively respond to a single stress factor; for instance, transcription levels of MhGR-RBP1 increase in response to drought, high salt, hydrogen peroxide, and nociceptive stress. This suggests that GR-RBPs have the potential to mediate responses to multiple stressors.

Interestingly, GR-RBPs also play roles in regulating plant growth and development under non-stress conditions. They influence processes such as leaf development and senescence, flowering, and fruit ripening. Moreover, the function of GR-RBPs during plant growth and development is not limited to specific tissues but rather has a global impact, as GR-RBPs are typically expressed throughout the plant. Changes in GR-RBPs can lead to simultaneous effects on different plant tissues. For example, the partial loss of RBP-P and RBP-L function in rice results in a wide range of phenotypic variations, including dwarfism, chlorophyll deficiency, sterility, delayed flowering, and reduced spikelet fertility [23].

With the increasing frequency of extreme weather events and natural disasters, the development of new stress-resistant crop varieties has become an urgent priority. We firmly believe that GR-RBPs will play a pivotal role in regulating plant growth and development in response to environmental stress. Consequently, it is imperative to conduct further exploration to identify additional crop varieties with GR-RBPs. However, compared to *Arabidopsis thaliana*, there has been limited research on the regulatory mechanisms of GR-RBPs in crops, partly due to the extended time periods involved and technical challenges. While the functional mechanisms of GR-RBPs in plants have primarily been studied in terms of their search for target RNAs and metabolic regulation [85], it is worth noting that in animals, RBPs have been implicated in RNA structure formation and can also be regulated by RNA structure [113]. Therefore, the pursuit of more crop varieties with GR-RBPs and a deeper investigation into the functional mechanisms of GR-RBPs in crops to mitigate stress represent crucial breeding objectives for enhancing crop yields in adverse environments.

## Figures and Tables

**Table 1:** Summary of glycine-rich RNA binding proteins in grain crops.

Gene	Plant Species	Roles	Phenotype	Reference
*CqGR-RBP2*	*Chenopodium quinoa*	Drought resistance	Not determined	[88]
*HvGR-RBP1*	*Hordeum vulgare*	Regulates the timing of anthesis and leaf senescence; cold resistance	Not determined	[89]
*HvGRP3*	*Hordeum vulgare*	Fungal pathogens and cold stress induce	Not determined	[90]
*MA16*	*Zea mays*	rRNA metabolism	Not determined	[80,81,82,83]
*OsRBP-P OsRBP-L*	*Oryza sativa*	Correct localization of mRNA	The *rbp-l/rbp-p* knockdown mutant exhibits plant dwarfism, chlorophyll deficiency, sterility to late flowering, and low spikelet fertility	[23,76]
*OsGRP1*	*Oryza sativa*	Enhances cell elongation; cold resistance	Overexpression of *OsGRP1* in Arabidopsis suppresses the dwarf phenotype of the mutant *bri1-5;* recovery cold adaptation to cold-sensitive E. coli mutant strains	[30,69,73]
*OsGRP2*	*Oryza sativa*	Cell wall construction; floral organ early development	Not determined	[74]
*OsGRP3*	*Oryza sativa*	Drought resistance; mRNA stability	Overexpression of *OsGRP3* in rice had better growth and higher rate after drought treatment, while transgenic plants with *OsGRP3* knocked out or knocked down had lower survival rate.	[71]
*OsGRP4*	*Oryza sativa*	Cold resistance; Heat resistance; mRNA export;	Under low temperatures, promotes seed germination and seedling growth	[72]
*OsGRP6*	*Oryza sativa*	Cold resistance; mRNA export	Recovery cold adaptation to cold-sensitive E. coli mutant strains	[69]
*OsRZ-2*	*Oryza sativa*	Cold resistance	The overexpression of *OsRZ-2* restore the cold-sensitive phenotype of *grp7* mutants	[70]
*SbGR-RNP*	*Sorghum bicolor*	Salinity- and ABA-induced upregulation; light response	Not determined	[91]
*TaGRP2*	*Triticum aestivum*	Flowering repressors	The flowering was delayed in overexpression *TaGRP2* plants and accelerated in RNAi-*TaGRP2* plants	[78]
*TaRZ1*	*Triticum aestivum*	Cold resistance; salt resistance	Overexpression *TaRZ1* in Arabidopsis delays seed germination and inhibits seedling growth under salt stress, and seedling growth was inhibited at low temperature.	[53]
*TaRZ2*	*Triticum aestivum*	Cold resistance; salt resistance	Overexpression *TaRZ2* in Arabidopsis delays seed germination under salt and drought stress and enhances the freezing tolerance.	[53]
*TaRZ3*	*Triticum aestivum*	Cold resistance	Overexpression *TaRZ3* in Arabidopsis delays seed germination under salt and drought stress	[53]
*whGRP-1*	*Triticum aestivum*	ABA-inducible gene	Not determined	[77]
*ZmGRP1*	*Zea mays*	Photoperiodic mRNA splicing and export	Not determined	[86]
*ZmGRP2*	*Zea mays*	Insect and fungal infection resistance	Upregulation of *ZmGRP2* can help maize to better fight against *Meloidogyne arenaria* infection.	[85]

**Table 2:** Summary of glycine-rich RNA binding proteins in horticultural crops.

Gene	Plant Species	Roles	Phenotype	Reference
*BoCSDP5*	*Brassica oleracea*	Cold resistance	Not determined	[51]
*BnGRP1*	*Brassica napus*	Cold resistance	Accelerated germination by overexpression *BnGRP1* in Arabidopsis	[99]
*CsGR-RBP3*	*Cucumis sativa*	Cold resistance	Arabidopsis plant overexpressing *CsGR-RBP3* shows strong cold tolerance at 0 °C and −20 °C	[98]
*LeRBP1*	*Solanum lycopersicum*	Cold resistance	Not determined	[96]
*MpGR-RBP1*	*Malus prunifolia*	Salt stress tolerance; oxidative stress tolerance	Overexpression MpGR-RBP1 in Arabidopsis accelerates seed germination and seedling growth when plants were exposed to high salt or oxidative stress	[101]
*SlORRM4*	*Solanum lycopersicum*	Mitochondrial RNA editing	The *slorrm4* mutant shows delayed fruit ripening	[94]
*SlRZ1A-like*	*Solanum lycopersicum*	Regulates target RNA transcription and translation	The *rzla-l* mutant fruit became smaller and ultimately less red in color	[95]
*SlRBP1*	*Solanum lycopersicum*	Regulates correct translation of target RNA; chloroplast development	The *slrbp1* knockdown mutant exhibits dwarf tomato plants with yellow leaves, smaller flowers, and fruit	[68]
*VviGRP2, VviRZ-1A* *VviGRP3* *VviGRP5 VviGRP7*	*Vitis vinifera*	Vitis vinifera mesocarp development	Not determined	[97]

**Table 3:** Summary of glycine-rich RNA binding proteins in other crops.

Gene	Plant Species	Roles	Phenotype	Reference
*CsGRP2s*	*Camelina sativa*	Cold resistance; RNA chaperone activity	Overexpression *CsGRP2s* had the ability to complement cold-sensitive *Escherichia coli* mutants at low temperatures	[14]
*CsGRP7a*	*Camelina sativa*	Cold resistance; RNA chaperone activity	Overexpression *CsGRP7a* in camelina grows sluggishly under salt stress, but its root grows better under cold stress than WT	[106]
*LbGRP1*	*Limonium bicolor*	Salt resistance	Overexpression *LbGRP1* in tobacco significantly improved superoxide dismutase, catalase activities, and proline levels under salt-stress conditions	[107]
*NgRBP*	*Nicotiana glutinosa*	Inhibition of RNA silencing; viral resistance	Knockdown of *NgRBP* enhanced resistance to PVX and cucumber mosaic virus	[105]
*NbRZ-1A*	*Nicotiana benthamiana*	mRNA alternative splicing; pathogen resistance	Not determined	[104]
*NbGRP7*	*Nicotiana benthamiana*	Participating in plant effector-triggered immunity	Mutation or ectopic expression of the *NbGRP7* compromises Rx1-mediated defense	[103]
*PpGRPs*	*Physcomitrella patens*	Low temperature response	Not determined	[109]
*SaRBP1*	*Suaeda asparagoides*	Salt resistance	Overexpression *SaRBP1* Arabidopsis seedlings displays longer primary roots, more fresh weight, higher number of lateral roots, and higher survival rates than WT	[108]
*ZjGRP*	*Zoysia japonica*	Salt resistance	Overexpression of *ZjGRP* increases resistance to salt stress in Arabidopsis	[110]

## Data Availability

Not applicable.
